# Cardiovascular reactivity to acute stress associated with sickness absence among Japanese men and women: A prospective study

**DOI:** 10.1002/brb3.1541

**Published:** 2020-02-20

**Authors:** Kumi Hirokawa, Tetsuya Ohira, Mitsugu Kajiura, Hironori Imano, Akihiko Kitamura, Masahiko Kiyama, Takeo Okada, Hiroyasu Iso

**Affiliations:** ^1^ Department of Nursing Baika Women's University (Hirokawa) Ibaraki Osaka Japan; ^2^ Public Health, Department of Social Medicine Osaka University Graduate School of Medicine Suita Osaka Japan; ^3^ Department of Epidemiology Fukushima Medical University School of Medicine Fukushima Japan; ^4^ Osaka Center for Cancer and Cardiovascular Disease Prevention Osaka Japan; ^5^ Tokyo Metropolitan Institute of Gerontology Tokyo Japan

**Keywords:** autonomic reactivity to stress, heart rate variability, Japanese workers, mental problems, prospective study, sickness absence

## Abstract

**Objective:**

We aimed to investigate associations between cardiovascular reactivity to acute stress and sickness absence among Japanese male and female workers, in a prospective study.

**Methods:**

Among healthy employed Japanese workers who underwent mental health checks between 2006 and 2009, data of 111 participants were analyzed. Changes in blood pressure, pulse rate, peripheral blood flow (PBF), and heart rate variability (HRV) (high frequency [HF] and low frequency [LF]) were calculated using differences between the two tasks, mirror drawing stress [MDS] and a maze task, and the postperiod value. Sickness absence through March 2010 was followed up by mail survey (average follow‐up 2.3 years). Logistic regression analysis was used, adjusting for lifestyle factors.

**Results:**

Among 12 participants who took sickness absences, eight were owing to mental problems. Changes in the LF during the MDS and maze tasks and LF‐to‐HF ratio during the MDS task were positively associated with all sickness absences (odds ratio [OR], 95% confidence interval [CI]: 2.09, 1.03–4.22; 2.04, 1.09–3.82; and 3.10, 1.46–6.58, respectively). Changes in PBF during the MDS task were also associated with increased risk of sickness absence (OR, 95% CI: 2.53, 1.10–5.81).

**Conclusion:**

Cardiovascular reactivity to acute stress should be considered at workers' health checks.

## Graphical abstract

Changes in the LF during the MDS and maze tasks and LF‐to‐HF ratio during the MDS task were positively associated with all sickness absences. Changes in PBF during the MDS task were also associated with increased risk of sickness absence.

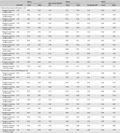

## 1 INTRODUCTION

Sickness absence due to mental illness such as depressive disorders and physical illness such as musculoskeletal pain has become a considerable problem (Kristiansen et al., 2011; Takada, Ebara, & Kamijima, 2010). Stress at work has been revealed as having an important role in sickness absence (Kivimäki et al., 2010; Mather, Bergström, Blom, & Svedberg, 2015; Theorell et al., 2015). Perceived psychological stress levels at work have been investigated, based on a conceptual model, to predict sickness absence, for example, the Job Demand–Control model (Karasek, 1979), the Effort–Reward Imbalance model (Siegrist, 1998), and the Organizational Justice model (Moorman, 1991). However, these perceived psychological stress levels are a theory‐based operationalization of stress at work. It is of interest to examine psychophysiological biomarkers for sickness absence.

Cardiovascular stress reactivity is a predictor of atherosclerosis and cardiac events (Gianaros et al., 2005). A meta‐analysis study on the associations between cardiovascular responses to laboratory stress and risk of cardiovascular disease revealed that greater reactivity to acute mental stress has an adverse effect on future cardiovascular risk status (Chida & Steptoe, 2010). Heightened blood pressure owing to mental stress has been associated with risk of hypertension over an 8‐year period among British civil servants from the Whitehall II cohort study (Steptoe, Kivimäki, Lowe, Rumley, & Hamer, 2016). Heart rate variability (HRV) is a widely used measure for studying cardiac autonomic modulation of the heart rate (Taylor, 2010). The vagus nerve has been known to play a significant role in health and disease, and decreased cardiac vagal tone measured by HRV has been associated with stress at work (Jarczok et al., 2013). Two review studies on stress at work and HRV showed negative associations between stress at work and HRV components (Chandola, Heraclides, & Kumari, 2010; Jarczok et al., 2013). Furthermore, according to another review study (Sgoifo, Carnevali, Alfonso Mde, & Amore, 2015), decreased HRV is not only a valuable marker of cardiovascular morbidity and mortality risk but it is also linked with other physical dysfunctions as well as a number of psychopathological conditions, including depression. HRV is one of the objective indices of autonomic nerve balance that is often related to physiological and psychological responses to stressors (Takada et al., 2010).

A few studies have investigated cardiovascular reactivity to stress in workers who had sickness absence. Although one previous case–control study showed no association between resting blood pressure and sickness absence (Takada et al., 2010), another case–control study demonstrated elevated basal systolic blood pressure (SBP) in males with sickness absence owing to job burnout, in comparison with healthy controls (de Vente, van Amsterdam, Olff, Kamphuis, & Emmelkamp, 2015). As for HRV, lower high frequency (HF) was found in workers who had sickness absence owing to depressive disorders (Takada et al., 2010) and burnout syndrome (de Vente et al., 2015), compared with healthy controls. Higher low frequency (LF) and higher LF‐to‐HF ratio were also found among workers who had sickness absence as a result of depressive disorders (Takada et al., 2010). In comparison with workers who had short‐term sickness absence, lower HF and also lower LF were found in workers with long‐term sickness absence (Kristiansen et al., 2011). To our best knowledge, no prospective study has investigated whether cardiovascular reactivity to stress is predictive for sickness absence.

The purpose of this prospective study was to investigate the associations between cardiovascular reactivity to acute stress and sickness absence among Japanese male and female workers. We hypothesized that higher blood pressure reactivity, higher LF and lower HF, and higher LF‐to‐HF ratio may be predictive for increased risk of sickness absence.

## 2 METHODS

### 2.1 Participants

In the present study, 111 (39 men, 72 women) participants were included in the analysis. Table 1 shows participants' measured variables at baseline. Compared with the excluded participants, participants included in the analysis were older and had greater change in HF.

**Table 1:** Participants' characteristics at baseline

	Analyzed participants	Excluded participants	*p*
*N*	111	86	
Age (years) (Mean ± *SD*)	48.6 ± 7.9	46.15 ± 8.6	.039
Depressive symptoms (Mean ± *SD*)	17.0 ± 9.5	16.0 ± 10.4	.48
Changes in SBP at MDS (mmHg) (Mean ± *SD*)	12.6 ± 12.1	12.3 ± 10.7	.84
Changes in SBP at Maze (mmHg) (Mean ± *SD*)	10.4 ± 9.7	9.5 ± 8.6	.53
Changes in DBP at MDS (mmHg) (Mean ± *SD*)	9.2 ± 8.7	9.4 ± 7.2	.88
Changes in DBP at Maze (mmHg) (Mean ± *SD*)	5.9 ± 6.0	5.7 ± 5.8	.75
Changes in PR at MDS (beats/min) (Mean ± *SD*)	0.9 ± 5.1	0.2 ± 4.5	.38
Changes in PR at Maze (beats/min) (Mean ± *SD*)	0.7 ± 4.1	0.1 ± 4.0	.31
Changes in PBF at MDS (PU) (Mean ± *SD*)	−33.7 ± 51.2	−26.8 ± 38.8	.29
Changes in PBF at Maze (PU) (Mean ± *SD*)	−26.1 ± 48.2	−16.9 ± 32.2	.11
Changes in LF at MDS (ms^2^/Hz) (Mean ± *SD*)	15.5 ± 64.4	22.5 ± 58.9	.44
Changes in LF at Maze (ms^2^/Hz) (Mean ± *SD*)	9.4 ± 75.4	16.4 ± 51.6	.47
Changes in HF at MDS (ms^2^/Hz) (Mean ± *SD*)	−37.2 ± 45.4	−25.1 ± 27.3	.021
Changes in HF at Maze (ms^2^/Hz) (Mean ± *SD*)	−35.6 ± 49.2	−23.1 ± 29.5	.029
Changes in LF/HF at MDS (Mean ± *SD*)	1.7 ± 1.5	2.1 ± 2.0	.10
Changes in LF/HF at Maze (Mean ± *SD*)	1.5 ± 1.5	1.7 ± 1.5	.43
Women (%)	64.9	67.4	.71
Manager/professional (%)	76.6	75.6	.87
Smoker (%)	9.0	17.4	.078
Alcohol drinking habit (%)	51.4	59.3	.27
Physically active (%)	57.7	48.8	.22
Obesity (BMI ≥ 25) (%)	20.7	19.8	.87
Past history (%)	52.3	43.0	.20

Note

Mean and *SD* of HRV for excluded participants were calculated based on 83 participants without missing values. Based on a *t* test to compare analyzed and excluded participants for continuous variables and on a chi‐square test for categorized variables.

The present study was conducted after obtaining approval from the Ethics Committee of the Osaka Medical Center for Health Science and Promotion.

### 2.2 Questionnaires and measurements

#### 2.2.1 Demographics

Participants were administered a questionnaire when they enrolled in the present study. They were asked about their sex, age, and current work. Occupational status was categorized as manager/professional and general worker. Teachers were categorized as professionals, based on previous studies and vital statistics in Japan (Fukuda, Nakamura, & Takano, 2005; Saeki, Hiroko, & Sakata, 2000). Participants were asked about their smoking status (categorized as current smoker or never/ex‐smoker) and alcohol consumption status (categorized as current drinker or never/ex‐drinker). The scale of the Japan Arteriosclerosis Longitudinal Study was used to evaluate physical activity (Naito et al., 2003). Participants were asked whether they exercised regularly for >15 min within the previous 3 months; individuals were categorized as physically active if they answered “Yes.” Depressive symptoms were assessed using the Japanese translation of the Center for Epidemiologic Studies Depression Scale (CES‐D scale) (Radloff, 1977; Shima, Shikano, Kitamura, & Asai, 1985), of which 20 items (alpha: 0.88) were scored from 0 (not at all) to 3 (for > 5 days).

#### 2.2.2 Medical history

Information on medication was queried, and use of medication for antihypertension, diabetes mellitus, or hyperlipidemia was identified in the questionnaires. Hypertension was defined as systolic blood pressure ≥140 mmHg and/or diastolic blood pressure ≥90 mmHg and/or use of antihypertensive medication. Diabetes mellitus was defined as fasting glucose level of ≥ 126 mg/dl or nonfasting glucose level of ≥200 mg/dl, and/or the use of medication for diabetes mellitus. Hyperlipidemia was defined as total cholesterol level ≥220 mg/dl and/or use of medication for hyperlipidemia. Participants with a medical history of such conditions were categorized as having hypertension and/or diabetes mellitus and/or hyperlipidemia. No participant had a history of stroke or myocardial infarction.

#### 2.2.3 Anthropometrics

Height in feet (in stockings or socks) and weight in light clothing were measured during the mental health checkups, and body mass index was calculated (kg/m^2^). Blood pressure was measured and peripheral blood collected between 2 p.m. and 4 p.m. at the front desk of the mental health facility.

Cardiovascular reactivity to stress was measured in an experimental room. After electrodes and apparatus were placed on participants, SBP (mmHg) and diastolic blood pressure (DBP: mmHg) were measured using a tonometry method, which has a low artifact rating and high accuracy of blood pressure measurement during reactivity testing (Nelesen & Dimsdale, 2002). Pulse rate (PR: beats/min) was measured with electrocardiography (BP‐508SD: Omron Colin, Tokyo, Japan) for 2 min as a pretask interval. Electrocardiograms were monitored from electrodes on the left subclavicular area and right lower chest. R–R intervals were measured using MemCalc (GMS Co., Ltd., Japan), which analyzes data while eliminating abnormal cardiac rhythms. If persistent atrial fibrillation was present and/or >10% of the recorded cardiac rhythm was abnormal, those data were omitted from the analyses. Power spectral analyses for R–R intervals on electrocardiograms were conducted every 128 beats to yield low‐frequency (LF) (0.04–0.15 Hz) and high‐frequency (HF) components (0.15–0.40 Hz) of HRV and their ratio (LF to HF).

Peripheral blood flow (PBF), in laser Doppler perfusion units (PU), was measured on the third finger using a laser Doppler blood flowmeter (PriFlux PF‐4000; PERIMED, Sweden) during the experimental periods. PBF was obtained from the product of the number of red blood cells and blood flow velocity approximately 0.5 mm below the skin surface (Wardell, Jakobsson, & Nilsson, 1993; Yamada & Ohta, 2005).

#### 2.2.4 Sickness absence

Information about sickness absence was collected by asking whether a participant had been absent from work owing to illness since the mental health checkups. If participants responded “Yes,” they were also asked to indicate the date of their absence. If participants had several instances of sickness absence, they were instructed to indicate the date of the first absence since their mental health checkup. They were asked the reason(s) for absence: physical illness and/or mental illness and/or other reasons, such as caring for aged parents.

### 2.3 Experimental tasks and procedure

#### 2.3.1 Enrollment of participants

Participants were enrolled in a “physical and mental refreshing course” comprising mental health checkups, which was conducted through the Osaka Medical Center for Health Science and Promotion between 2006 and 2009. The mental health checkups were applied through companies in the Osaka region, as well as on the Osaka Medical Center for Health Science and Promotion website. A total of 551 Japanese workers (212 men, 339 women; age range: 21–73 years) underwent the abovementioned at baseline. Among them, the most frequently reported occupation was teacher (54% of men, 67% of women). Because enrollment was promoted via health insurance societies in Osaka, including the Mutual Aid Association of Public School Teachers, most participants were teachers. The data from the mental health checkups have been reported elsewhere (Hirokawa et al., 2014, 2016a, 2016b).

Sickness absence data of the 197 (67 men, 130 women) participants who gave their written consent to participate in the study, from the date of their mental health checkup through 31 March 2010, were followed up using a mailed questionnaire. Among the 197 participants, 119 individuals returned completed questionnaires (response rate: 60.4%), and 115 (40 men, 75 women) reported sickness absence. However, three respondents had taken absence before their mental health checkup, and HRV data were missing for one respondent; therefore, these participants were excluded from the analyses. Finally, 111 respondents were included in the analyses (effective response rate: 56.3%).

#### 2.3.2 Procedure

At the mental health checkups, participants were administered questionnaires, anthropometric measurement, blood sample collection, and experimental tasks.

Participants had to do two experimental tasks comprised of a modified version of a mirror drawing stress (MDS) task and a maze task. These tasks have been widely used to examine cardiovascular reactivity in a laboratory setting (Owens, Stoney, & Matthews, 1993; Sato & Miyake, 2004). In the MDS task, a complex pathway is presented on a computer screen for 2 min, and participants are asked to “trace” the pathway with a computer mouse as accurately and as rapidly as possible (Hirokawa et al., 2014, 2016a, 2016b). The maze task (Amthat: Brain Medical, Tokyo, Japan), which is designed to assess perceptual functioning (especially thinking ability), is also presented on a computer screen for 2 min. Participants are required to study the maze and plan how to reach the goal by passing through “invisible” walls in a grid comprising five lines and five columns (Hirokawa et al., 2014, 2016a, 2016b).

After the two experimental tasks were finished, participants' cardiovascular reactivity to stress was measured for 2 min as a post‐task interval. The sequence of these tasks was fixed as “pretask interval,” “MDS task,” “maze task,” and “post‐task interval.”

### 2.4 Statistical methods

The average HRV during the two tasks was computed. Because measured variables during pretask periods were higher than those during post‐task periods, changes between values during the task (MDS and maze tasks) and the post‐task as the rest period were calculated as recovery from a stressful task (Hirokawa et al., 2014). Standard scores (*z*‐scores) of changes were calculated (minus the mean score and divided by the standard deviation). We calculated the crude odds ratio (OR) and 95% confidence interval (95% CI) for sickness absence according to the *z*‐score of cardiovascular reactivity to stress, and age‐ and sex‐adjusted ORs, using a logistic regression model. The fully adjusted ORs and 95% CIs for sickness absence according to the *z*‐score of cardiovascular reactivity to stress were also computed, adjusting for age, sex, body mass index, occupational position, depressive symptoms, smoking status, alcohol consumption, physical activity, and medical history. All statistical analyses were performed with IBM SPSS Statistics for Windows, version 24 (IBM Corp.).

## 3 RESULTS

During the mean 2.3 years (standard deviation: 0.9, range: 0.02–3.69) of follow‐up of 111 healthy employees (255.9 person‐years), 12 (4 men, 8 women) took sickness absence for any reason; among them, 8 (3 men, 5 women) took sickness absence for reasons of mental illness.

Table 2 shows crude ORs, age‐ and sex‐adjusted ORs, and fully adjusted ORs in relation to sickness absence owing to all causes. Changes in LF during both tasks were associated with increased risk of all sickness absence (fully adjusted OR: 2.09, 95% CI: 1.03–4.22 for MDS task, and 2.04, 95% CI: 1.09–3.82 for maze task). Changes in the LF‐to‐HF ratio during the MDS task were associated with increased risk of all sickness absence (fully adjusted OR: 3.10, 95% CI: 1.46–6.58). Changes in SBP, DBP, and PR were not associated with sickness absence. However, changes in PBF during the MDS task were significantly associated with increased risk of all sickness absence (fully adjusted OR: 2.53, 95% CI: 1.10–5.81).

**Table 2:** Associations between changes in reactivity of heart rate variability and sickness absence due to all causes and mental health

	Crude OR	95% CI		Age‐ and sex‐adjusted OR	95% CI		Full adjusted OR^a^	95% CI	
		Lower	Upper		Lower	Upper		Lower	Upper
Sickness absence due to all causes (*n* = 12)									
Changes in reactivity of SBP at MDS	1.30	0.74	2.29	1.29	0.71	2.34	1.19	0.62	2.30
Changes in reactivity of SBP at Maze	1.02	0.56	1.87	1.11	0.57	2.13	1.00	0.50	2.00
Changes in reactivity of DBP at MDS	1.35	0.81	2.27	1.29	0.75	2.21	1.22	0.67	2.22
Changes in reactivity of DBP at Maze	1.20	0.67	2.15	1.22	0.65	2.28	1.12	0.56	2.22
Changes in reactivity of PR at MDS	1.03	0.57	1.88	1.11	0.62	2.00	1.21	0.67	2.20
Changes in reactivity of PR at Maze	1.06	0.59	1.93	1.17	0.64	2.15	1.24	0.64	2.42
Changes in reactivity of PBF at MDS	2.00	0.98	4.05	2.06	0.99	4.30	2.53	1.10	5.81
Changes in reactivity of PBF at Maze	1.17	0.64	2.13	1.19	0.66	2.14	1.24	0.67	2.27
Changes in reactivity of LF at MDS	1.89	1.02	3.52	1.82	0.97	3.42	2.09	1.03	4.22
Changes in reactivity of LF at Maze	1.68	0.96	2.92	1.72	0.98	3.02	2.04	1.09	3.82
Changes in reactivity of HF at MDS	0.71	0.42	1.18	0.72	0.43	1.22	0.68	0.39	1.20
Changes in reactivity of HF at Maze	0.88	0.53	1.48	0.91	0.52	1.57	0.94	0.54	1.65
Changes in reactivity of LF/HF at MDS	1.81	1.07	3.07	2.11	1.18	3.76	3.10	1.46	6.58
Changes in reactivity of LF/HF at Maze	1.27	0.73	2.20	1.34	0.76	2.37	1.33	0.75	2.37
Sickness absence due to mental illness (*n* = 8)									
Changes in reactivity of SBP at MDS	0.93	0.45	1.95	0.90	0.43	1.90	0.67	0.30	1.48
Changes in reactivity of SBP at Maze	0.70	0.32	1.50	0.71	0.31	1.60	0.61	0.26	1.47
Changes in reactivity of DBP at MDS	0.98	0.47	2.03	0.90	0.43	1.90	0.72	0.35	1.47
Changes in reactivity of DBP at Maze	0.84	0.41	1.74	0.82	0.38	1.79	0.70	0.32	1.51
Changes in reactivity of PR at MDS	1.17	0.59	2.35	1.27	0.63	2.53	1.43	0.66	3.08
Changes in reactivity of PR at Maze	1.31	0.65	2.63	1.47	0.70	3.06	1.44	0.62	3.32
Changes in reactivity of PBF at MDS	2.66	1.08	6.56	2.73	1.08	6.93	3.70	1.17	11.64
Changes in reactivity of PBF at Maze	1.25	0.61	2.57	1.25	0.62	2.52	1.37	0.66	2.85
Changes in reactivity of LF at MDS	1.41	0.69	2.90	1.36	0.66	2.80	1.53	0.67	3.49
Changes in reactivity of LF at Maze	1.66	0.91	3.05	1.72	0.92	3.21	2.06	0.99	4.27
Changes in reactivity of HF at MDS	1.14	0.52	2.50	1.17	0.53	2.58	1.09	0.48	2.47
Changes in reactivity of HF at Maze	1.22	0.49	3.06	1.29	0.50	3.31	1.23	0.49	3.12
Changes in reactivity of LF/HF at MDS	1.84	1.03	3.29	2.06	1.09	3.88	3.56	1.44	8.80
Changes in reactivity of LF/HF at Maze	1.54	0.83	2.83	1.62	0.86	3.06	1.69	0.84	3.39

Note

Changes in reactivity were transformed as standard scores (*z*‐scores).

a Age, sex, occupational position, overweight, medical history, depressive symptoms, smoking, alcohol drinking, and physical activity were adjusted.

Associations of changes in the LF‐to‐HF ratio during the MDS task with sickness absence were robust, when limited to sickness absence owing to mental illness: (fully adjusted OR: 3.56, 95% CI: 1.44–8.80). On the other hand, associations between changes in LF and sickness absence owing to mental illness become nonsignificant (Table 2). Changes in PBF during the MDS task were also robustly associated with increased risk of sickness absence owing to mental illness (fully adjusted OR: 3.70, 95% CI: 1.17–11.64).

In the present study, we included five participants with a past history of mental illness. When those participants were excluded from the analyses, 11 (3 men, 8 women) participants took sickness absence; among these, 7 (2 men, 5 women) took sickness absence for reasons of mental illness. The associations between HRV and sickness absence were the same: LF during both tasks (fully adjusted OR: 2.18, 95% CI: 1.07–4.43 for MDS task and 1.92, 95% CI: 1.02–3.62 for maze task), LF‐to‐HF ratio during the MDS task (OR: 3.15, 95% CI: 1.45–6.83) with all sickness absence, and LF‐to‐HF ratio during the MDS task (OR: 3.51, 95% CI: 1.41–8.75) with sickness absence owing to mental illness. Changes in PBF during the MDS task were also robustly associated with increased risk of all sickness absence (OR: 3.07, 95% CI: 1.19–7.94) and with increased risk of sickness absence owing to mental illness (OR: 4.28, 95% CI: 1.26–14.57).

## 4 DISCUSSION

In the present study of healthy employed Japanese workers, changes in the LF and LF‐to‐HF ratio, especially during the MDS task, were positively associated with sickness absence from all causes. Although changes in blood pressure and pulse rate were not associated with sickness absence, changes in peripheral blood flow during the MDS task were also positively associated with sickness absence from all causes. When limited to sickness absence owing to mental illness, changes in the LF‐to‐HF ratio during the MDS task were also positively associated with sickness absence for mental health reasons. Changes in the PBF during the MDS task were also robustly associated with sickness absence owing to mental illness. Changes in the HRV and PBF during the MDS task could be an objective predictor for sickness absences from work.

The results of the present study revealed the same trend as in a previous case–control study (Takada et al., 2010), which compared blood pressure and HRV between workers with sickness absence owing to depressive disorders and healthy controls. The present study had 12 participants with sickness absence, and eight of them were absent because of mental illness. The HRV components LF and LF‐to‐HF ratio may be reflective of mental health status. The HRV component LF is influenced by both sympathetic and parasympathetic activities (Billman, 2013). The LF‐to‐HF ratio has been proposed as a measure of sympathetic/parasympathetic activity balance (Pagani et al., 1988). It has been reported that dominant sympathetic nerve activity reflects an increase in LF and/or a decrease in the HF component of HRV (Rechlin, Weis, Spitzer, & Kaschka, 1994). Dominant sympathetic nerve activity, which reflects an increase in the LF and/or a decrease in the HF component, could be an objective index for predicting sickness absence.

Most sickness absences in the present study were because of mental illness. Low HRV has been implicated in a variety of psychiatric diseases, not only in depression (Sgoifo et al., 2015) but also in bipolar disorder (Faurholt‐Jepsen, Kessing, & Munkholm, 2017) and schizophrenia (Clamor, Lincoln, Thayer, & Koenig, 2016). Reductions in HF variability (parasympathetic modulation), which were consistently higher LF‐to‐HF ratio, were found in patients with those psychiatric diseases compared to healthy controls; this suggests dysfunctions on the activity of the autonomic nervous system and a sympathetic prevalence (Sgoifo et al., 2015). Harrison, Cooper, Voon, Miles, and Critchley (2013) suggested that depression‐associated alterations in the LF‐to‐HF ratio result from the effects of cytokines and inflammation of the central autonomic network. The effects of inflammation on blood pressure are mediated via a shift in the LF‐to‐HF ratio (Harrison et al., 2013). However, it is unclear whether reduced HRV in mental illness is caused by the symptoms of mental illness or the effects of treatment medications, such as antidepressants. A previous study pointed out the effects of antidepressants on reducing HRV (Licht, de Geus, van Dyck, & Penninx, 2010). In the present study, when participants with a past history of mental illness and those taking antidepressant medication were excluded from the analyses, the results did not change.

Furthermore, in the present study, changes in the LF‐to‐HF ratio were associated with increased risk of sickness absence, which was attributed to higher changes in the LF rather than HF component. The LF component increased in reaction to stressful tasks compared with a rest period, whereas the HF component decreased in reaction to stressful tasks compared with a rest period, as in our previous study (Hirokawa et al., 2014). During the rest period, both the HRV LF and HF components, as well as the LF‐to‐HF ratio, were not associated with risk of sickness absence (data were not shown); however, the LF component during the task periods was positively associated with risk of sickness absence. The Whitehall II longitudinal population‐based cohort study investigated associations between HRV at rest and perceived depressive symptoms at 10‐year follow‐up (Jandackova, Britton, Malik, & Steptoe, 2016). The results of that study revealed that higher HRV components at baseline were associated with lower depressive symptoms at follow‐up in men. Recovery from acutely stressful tasks may differ from HRV measurements at rest.

Changes in peripheral blood flow were also predictive for sickness absence. In the present study, the average change in PBF was lower with stressful tasks, and higher values of changes in the PBF (i.e., increased PBF or lower recovery from an acutely stressful task) were associated with increased risk of sickness absence. According to Hammadah et al. (2018), the sympathetic nervous system activation, which is induced by mental stress, increases myocardial oxygen demand and stimulates alpha‐adrenergic receptor‐mediated coronary constriction. Because regional blood flow responses are regulated by endothelium‐derived nitric oxide (Toda, 2012), in the presence of normal vascular endothelial function, vasoconstriction is countered by shear‐mediated release of nitric oxide and other endothelium‐dependent vasodilators (Hammadah et al., 2018). With endothelial dysfunction, adrenergic receptor‐mediated constriction supervenes, and the resulting coronary vasoconstriction limits appropriate coronary blood flow (Hammadah et al., 2018). Acute mental stress increases the coronary blood flow in people without coronary artery disease, in comparison with people who have coronary artery disease (Kop et al., 2001). Hammadah et al. (2018) revealed that coronary and peripheral blood flow responds similarly during a mental stress task. This is an important finding, demonstrating that the sympathetic nervous system activation is induced by acutely stressful tasks, especially overloading tasks such as the MDS task, and that lower cardiovascular reactivity to stressful tasks was predictive for future sickness absence.

There have been discussions whether increased or blunted cardiovascular responses to acute stress are associated with a health outcome. For example, physiological and psychological manipulations markedly raise sympathetic outflow but do not increase LF power, and often reduce it (Reyes del Paso, Langewitz, Mulder, van Roon, & Duschek, 2013). According to Reyes del Paso et al. (2013), the LF component was an index of sympathetic cardiac control and the LF‐to‐HF ratio was an index of autonomic balance, concluding that all HRV components are predominantly related to the parasympathetic system. The LF power was a poor index of sympathetic nervous reactivity to acute stress tasks but it was related to baroreflex function (reactivity to increased or decreased blood pressure in the carotid sinus) (Goldstein, Bentho, Park, & Sharabi, 2011). Accordingly, there are several studies showing that blunted cardiovascular reactivity to stress has been linked to increased depressive symptoms, risk of obesity, and poor self‐reported health status (Phillips, 2011; de Rooij, 2013). Whether blunted or high reactivity to stress is predictive for the risk of sickness absence is still not clear. According to a meta‐analysis on associations between cardiovascular reactivity to stress and risk of cardiovascular disease, greater reactivity to stress had an adverse effect on future cardiovascular risk (Chida & Steptoe, 2010). Some of the inconsistent results may be attributed to differences in study design (e.g., manipulations of stress tasks, duration of follow‐up, measurement of HRV), and confounding factors such as chronic illness and health behavior, which may affect cardiovascular reactivity to stress. Further studies should be conducted to clarify associations between cardiovascular reactivity to stress and sickness absence.

The present study had several strengths. We prospectively followed up participants who did not have a past history of stroke and myocardial infarction, until sickness absence. At the baseline survey, cardiovascular reactivity to mental stress was evaluated with various measures including blood pressure, heart rate, HRV, and PBF. Two different tasks were used for acute stress, to compare differences in reactivity that is dependent on a task. Furthermore, various potential confounders were considered in the analyses.

Other limitations in the present study should be discussed. First, the small number of participants did not permit us to analyze sex‐specific differences. The unrepresentative sample of Japanese working men and women could lead to biased results. Most participating professionals were teachers, so we could not consider occupational differences. Because participants were enrolled at the mental health checkups, they may be especially conscious of their mental health and/or may be a group at high risk for mental problems. Second, information on sickness absence was based on self‐reports received via post. We did not ask for details about diseases and reasons why participants took sickness absence, except for inquiring whether it was owing to physical or mental illness. The date of sickness absence may not be accurate depending on the participant's memory. Furthermore, follow‐up time differed depending on participants' enrollment. Because it can take long time to develop disease, participants should be followed up for a certain length of time. Finally, multiple factors may underlie perceived symptoms that lead to sickness absence. Other potential confounding factors, which were not measured in the present study, should be taken into consideration in future studies. For example, as discussed above, medication treatment for mental illness may affect HRV (Licht et al., 2010). As explained above, because there were many limitations in the present study, the results should be interpreted with caution. Owing to the problem of multiple testing using a small sample, there is greater likelihood of a chance finding.

Finally, absenteeism was the focus in the present study; however, there has also been much concern in recent studies about presenteeism. Presenteeism is another aspect of absenteeism, which refers to an employee who is present at work but is not actually productive owing to chronic ill health or personal problems. Presenteeism results in greater losses to an organization than those attributed to absenteeism (Stelzner, 2005). Mean annual costs per person for absenteeism were the highest in Japan, among eight countries including the United States, Canada, China, and Korea (Evans‐Lacko & Knapp, 2016). Mean costs per person for presenteeism were highest in South Africa (Evans‐Lacko & Knapp, 2016). Tsuchiya et al. (2012) found that there is a significant relationship between depression and lower rates of presenteeism, but there was no significant relationship between depression and absenteeism among Japanese workers. Evans‐Lacko and Knapp (2016) have pointed out that Asian countries have the lowest prevalence of diagnosed depression, which could be owing to a true difference or measurement bias. There may be cultural differences in relation to absenteeism and presenteeism (Evans‐Lacko & Knapp, 2016).

## 5 CONCLUSIONS

Changes in the LF‐to‐HF ratio and those in the PBF during an acutely stressful task were positively associated with increased risk of sickness absence from all causes, especially mental illness. The LF‐to‐HF ratio increased with stressful tasks, which may reflect sympathetic/parasympathetic activity balance. However, the PBF decreased with stressful tasks, which may reflect coronary circulation activated by the sympathetic nervous system. Cardiovascular stress reactivity could be an objective predictive factor for workers' physical and mental disorders in the workplace. Further studies are necessary to clarify associations between cardiovascular reactivity to acute stress and sickness absence.

## CONFLICT OF INTEREST

All authors report no conflicts of interest.

## AUTHOR CONTRIBUTION

Kumi Hirokawa analyzed the data and wrote this manuscript. Tetsuya Ohira engaged in manuscript writing and data analyses, and participated in data collection. Mitsugu Kajiura, Hironori Imano, Akihiko Kitamura, Masahiko Kiyama, and Takeo Okada participated in data collection and revised the manuscript. Hiroyuki Iso engaged in the conception of this manuscript writing and revised the manuscript.

## Data Availability

The data that support the findings of this study are available from the corresponding author upon reasonable request.
